# Association between *IL‐4* tagging single nucleotide polymorphisms and the risk of lung cancer in China

**DOI:** 10.1002/mgg3.585

**Published:** 2019-02-06

**Authors:** Nan Tan, Jiangjiang Song, Mengdan Yan, Jiamin Wu, Yao Sun, Zichao Xiong, Yipeng Ding

**Affiliations:** ^1^ Department of Cadre’s Ward Xi’an No.1 Hospital Xi’an Shaanxi China; ^2^ Key Laboratory of Resource Biology and Biotechnology in Western China (Northwest University), Ministry of Education Xi’an Shaanxi China; ^3^ Department of Emergency Hainan General Hospital Haikou Hainan P. R. China

**Keywords:** *interleukin‐4*, lung cancer, rs2227284, rs2243250

## Abstract

**Background:**

In China, lung cancer is also the most commonly diagnosed cancer with a lower 5‐year survival rate, leading to high social burdens. Recently, many studies highlighted the importance of inflammation in the initiation and progression of cancer. The goal of this study was to investigate the association between *interleukin‐4* (*IL‐4*, OMIM#147780) single nucleotide polymorphisms (SNPs) and lung cancer susceptibility.

**Methods:**

A case‐control study was conducted in a Chinese population including 199 male patients with lung cancer and 266 healthy men. Six SNPs selected from the HapMap database were genotyped using Agena MassARRAY. Genetic models and haplotype analyses were utilized to evaluate the association between SNPs and lung cancer risk.

**Results:**

In our findings, rs2243250 was associated with a decreased lung cancer risk under the log‐additive model (odds ratio, OR = 0.71, 95% confidence interval, CI = 0.51–0.97, *p* = 0.030), and the G/G genotype of rs2227284 conferred a negative effect; the risk of lung cancer under the codominant (OR = 0.19, 95% CI = 0.04–0.87, *p* = 0.040) and recessive models (OR = 0.20, 95% CI = 0.04–0.88, *p* = 0.012) after adjusted by age.

**Conclusions:**

These data indicated potential associations between *IL‐4* polymorphisms and lung cancer susceptibility. That may help to improve the understanding of the relationship between inflammation and lung cancer in the future.

## 1 INTRODUCTION

Currently, lung cancer is the most common cancer around the world and the leading cause of cancer‐related deaths (Stewart & Wild, 2014). In China, lung cancer is also the most commonly diagnosed cancer with a lower 5‐year survival rate, and the estimated age‐standardized mortality rate in 2015 for lung cancer was 610.2 per 100,000 population including 432.4/100,000 men and 177.8/100,000 women (Chen et al., 2016), which was much higher than the world average. According to the research, an estimated 733.3 per 100,000 new lung cancer patients occurred in China in 2015, including 509.5/100,000 males and 224.0/100,000 females (Chen et al., 2016). Comparing with the previous data, the incidence of lung cancer in China was on the rise, leading to high social and economic burdens (Hong et al., 2015). Therefore, it is necessary to enhance the efficiency of prevention and early diagnosis of lung cancer.

Many definite risk factors have been found to be linked to lung cancer risk, such as tobacco use, environmental pollution, food, genetics, and chronic obstructive pulmonary disease (Hong et al., 2015). Also, researchers revealed that genetic factors have an important role in the etiology of lung cancer, such as *TP53 *(OMIM#191170), *RB1* (OMIM# 614041), and so forth (Granville & Dennis, 2005; Yin et al., 2016). Recently, much attention of genetic susceptibility studies of cancer has been focused on single nucleotide polymorphisms (SNPs) in candidate genes. In 2010, Timofeeva et al. reported that polymorphisms in genes involved in Phase I and Phase II of xenobiotic metabolism are related to the risk of early‐onset lung cancer (Timofeeva et al., 2010). In 2015, Leng et al. conducted a genome‐wide association study, and they reported that a functional 15q12 variant was identified as a risk factor for lung cancer (Leng et al., 2015).

Many clinical and epidemiologic studies have suggested strong association between inflammation and lung cancer (Barreiro et al., 2013; Sohal, Mahmood, & Walters, 2014). Tumor cells could produce several cytokines and chemokines, some of which have been implicated to mediate different steps in the pathway leading to carcinogenesis (Gomes, Teixeira, Coelho, Araújo, & Rui, 2014). Interleukin‐4 (IL‐4) is an anti‐inflammatory cytokine related to the growth of some tumors, such as colon, breast, and lung (Gomes et al., 2012; Toi, Bicknell, & Harris, 1992). In lung cancer, *IL‑4* (OMIM#147780) polymorphisms have been associated with a reduced risk of non‐small cell lung cancer (NSCLC) among the Portuguese and Chinese populations (Gomes et al., 2012; Gu, Shen, & Zhang, 2014). However, the overall information about association between *IL‐4* polymorphisms and lung cancer risk is poor.

In this study, we performed a case‐control study to evaluate the association between SNPs distributed in the *IL‐4* gene and lung cancer susceptibility in a Chinese population. Six SNPs were genotyped and analyzed in an attempt to identify new susceptibility locus that may provide guidance for early diagnosis for lung cancer.

## 2 MATERIALS AND METHODS

### 2.1 Ethical compliance

The study protocol was abided by the Declaration of Helsinki and its later amendments or comparable ethical standards. Additionally, this study was approved by the Clinical Research Ethics of Hainan General Hospital for Approval of Research Involving Human Subjects and written informed consent was obtained from all individuals included in the study.

### 2.2 Study subjects and sample collection

A total of 199 male patients with lung cancer and 266 healthy men were included in this study. The demographic characteristics of the lung cancer patients recruited from the First Affiliated Hospital of Xi'an Jiao tong University were shown in Table 1. The inclusions of the case groups were: (a) males; (b) diagnosed with confirmed lung cancer patients; (c) did not have history of any other cancers; (d) never received chemotherapy or radiotherapy treatment before. Healthy controls with no evidence of lung or other cancers were selected from healthy men who did medical examination during the same period.

**Table 1:** Characteristics of the male individuals in cases and controls

	Group	*N*	Mean	Standard deviation (*SD*)	Mean ± SD	*p*‐value
Age	Case	199	59.31	9.642	59.31 ± 9.642	*p < *0.001*
	Control	266	48.06	12.555	48.06 ± 12.555	

* *p*‐values were calculated by Welch's *t* tests.

Five milliliters of venous blood samples were collected from each subject into tubes containing EDTA, then centrifuged and stored at −80°C.

### 2.3 SNP selection and genotyping

Six SNPs (rs2243250, rs2227284, rs2243267, rs2243270, rs2243283, rs2243289) with a minor allele frequency (MAF) > 0.05 in *IL‐4* gene (its GenBank reference is NC_000005.10) were selected from theHapMap database. Genomic DNA was extracted from blood sample by GoldMag‐Mini Whole Blood Genomic DNA Purification Kit (GoldMag Ltd. Xi'an. China) according to manufacturer's protocol, and the concentration of DNA was measured by Nanodrop 2000. MassARRAY Assay Design 3.0 Software (Agena, San Diego, CA, USA) was utilized to design Multiplex SNP MassEXTEND assays (Yang et al., 2005). SNPs genotyping were performed based on Agena MassARRAY RS1000 (Agena, Inc.) according to the manufacturer's protocol, and the Agena Typer Software, version 4.0 (Agena, Inc.) was used to manage and analyze the data as earlier described (Gabriel, Ziaugra, & Tabbaa, 2009; Thomas et al., 2007).

### 2.4 Statistical analysis

Data were analyzed using SPSS version 18.0 statistical software (SPSS Inc., Chicago, IL, United States) and Excel 20.0 (Microsoft Corp., Redmond, WA, United States) (Zhou et al., 2018). Distribution differences in age and gender between cases and controls were compared by using Welch's *t* test and Chi‐square test, respectively (Adamec, 1964). Hardy–Weinberg Equilibrium (HWE) was assessed for the frequency of each SNP using a goodness‐of‐fit χ^2^ test in the control subjects. Logistic regression analysis was used to determine the association between SNPs and the risk of lung cancer by calculating ORs and 95% CIs (Bland & Altman, 2000; Zheng et al., 2017). Web‐based software SNPStats (https://www.snpstats.net/start.htm?q=snpstats/start.htm) was used to analyze the association under five genetic models (codominant, dominant, over‐dominant, recessive, and log‐additive) with an adjustment of age (Valls & Iniesta, 2006). Haploview software package (version 4.2) and the SHEsis software platform were used to conduct linkage disequilibrium analysis and haplotype‐based associations (Barrett, Fry, Maller, & Daly, 2005; Shi & He, 2005). All *p* values in this study were two‐tailed, and *p* < 0.05 was considered statistically significant.

SNP–SNP interactions were investigated by using the MDR (Multifactor dimensionality reduction) software package (version 3.0.2). The best model with the maximization of cross‐validation consistency was selected (Ritchie et al., 2001). In addition, traditional statistical methods were performed to examine the results from MDR analyses, and *p* < 0.05 was considered statistically significant (Lin et al., 2013).

## 3 RESULTS

### 3.1 Demographic information of participants

A total of 199 patients with lung cancer and 266 healthy individuals were enrolled in this study, and all the participants are males. Demographic information of participants is listed in Table 1. The mean age of the participants was 48.06 ± 12.555 years in the control group and 59.31 ± 9.642 years in the case group. In addition, there were significant differences in age distribution between the case and control groups (*p* < 0.05).

### 3.2 Association between SNPs in *IL‐4* and lung cancer risk

Six SNPs (rs2243250, rs2227284, rs2243267, rs2243270, rs2243283, rs2243289) selected from the HapMap were genotyped, Table 2 summarized the frequency information of tested SNPs among the individuals in the case and control groups. In the control groups, all six SNPs were conformed to HWE (*p* > 0.05). Then, we conducted two‐sided Pearson chi‐square tests to identify differences in allele frequency distributions between cases and controls. As shown in Table 3, rs2243250 (HGVS: NM_000589.3:g.132673462C>T) was related to a decreased risk of lung cancer (OR = 0.692, 95% CI = 0.500–0.958, *p* = 0.026), and similar results (OR = 0.722, 95% CI = 0.521–1.001, *p* = 0.050) were observed in rs2243267 (HGVS: NM_000589.3: g.132678194G>C). No associations were observed between the other four SNPs and lung cancer. After Bonferroni correction, none of the SNPs showed statistically significant associations.

**Table 2:** Basic information of the SNPs in *IL‐4*

SNP ID	Position	Role	Allele	Case			Control			Case		Control	
			(A/B)	AA	AB	BB	AA	AB	BB	A (MAF)	B	A (MAF)	B
rs2243250	132,009,154	Promoter	C/T	7	57	135	19	89	158	0.178	0.822	0.239	0.761
rs2227284	132,012,725	Intron	G/T	2	49	148	13	66	186	0.133	0.867	0.174	0.826
rs2243267	132,013,886	Intron	G/C	7	57	135	17	89	160	0.178	0.822	0.231	0.769
rs2243270	132,014,109	Intron	A/G	7	57	134	17	89	160	0.179	0.821	0.231	0.769
rs2243283	132,016,593	Intron	G/C	7	55	137	14	76	175	0.173	0.827	0.196	0.804
rs2243289	132,018,132	Intron (boundary)	A/G	7	58	134	16	90	160	0.181	0.819	0.229	0.771

Note

SNP: single‐nucleotide polymorphism; MAF: minor allele frequency; A/B: minor/major alleles frequencies in the controls and cases.

The GenBank reference of *IL‐4*: NC_000005.10.

**Table 3:** *IL‐4* polymorphisms and lung cancer risk

SNP ID	HWE *p*‐value	ORs	95% CI	Pearson Chi‐square *p*‐value	*p‐*value after Bonferroni correction
rs2243250	0.236	0.69	0.500–0.958	0.026	0.156
rs2227284	0.050	0.73	0.507–1.055	0.093	0.558
rs2243267	0.387	0.72	0.521–1.001	0.050	0.300
rs2243270	0.387	0.73	0.524–1.007	0.054	0.324
rs2243283	0.170	0.86	0.614–1.203	0.376	1.000
rs2243289	0.489	0.74	0.536–1.028	0.072	0.432

Note

SNP: single‐nucleotide polymorphism; HWE: Hardy–Weinberg equilibrium; ORs: odds ratios; 95% CI: 95% confidence interval.

The GenBank reference of *IL‐4*: NC_000005.10.

*p*‐values were calculated by Pearson χ^2^ test.

Additionally, we assessed the association under five different genetic models (dominant, recessive, log‐additive, codominant, and over‐dominant) by logistic regression analysis. As shown in Table 4, rs2243250 was associated with a decreased lung cancer risk under the log‐additive model (OR = 0.71, 95% CI = 0.51–0.97, *p* = 0.030); the G/G genotype of rs2227284 (HGVS: NM_000589.3:g.132677033T>G) conferred a protective effect on the lung cancer risk in the codominant (OR = 0.19, 95% CI = 0.04–0.87, *p* = 0.040); and recessive model (OR = 0.20, 95% CI = 0.04–0.88, *p* = 0.012). With adjustment by age, G/G genotype of rs2227284 still significantly decreased lung cancer risk by 0.20‐fold (OR = 0.20, 95% CI = 0.04–0.94, *p* = 0.020).

**Table 4:** Logistic regression analysis of the association between polymorphisms in *IL‐4* and lung cancer

SNP ID	Model	Genotype	Control	Case	OR (95% CI)	*p*‐value
rs2243250	Codominant	T/T	158 (59.4%)	135 (67.8%)	1.00	0.084
		C/T	89 (33.5%)	57 (28.6%)	0.75 (0.50–1.12)	
		C/C	19 (7.1%)	7 (3.5%)	0.43 (0.18–1.06)	
	Dominant	T/T	158 (59.4%)	135 (67.8%)	1.00	0.061
		C/T‐C/C	108 (40.6%)	64 (32.2%)	0.69 (0.47–1.02)	
	Recessive	T/T‐C/T	247 (92.9%)	192 (96.5%)	1.00	0.084
		C/C	19 (7.1%)	7 (3.5%)	0.47 (0.20–1.15)	
	Over‐dominant	T/T‐C/C	177 (66.5%)	142 (71.4%)	1.00	0.270
		C/T	89 (33.5%)	57 (28.6%)	0.80 (0.54–1.19)	
	Log‐additive	—	—	—	0.71 (0.51–0.97)	0.030
rs2227284	Codominant	T/T	186 (70.2%)	148 (74.4%)	1.00	0.040
		G/T	66 (24.9%)	49 (24.6%)	0.93 (0.61–1.43)	
		G/G	13 (4.9%)	2 (1%)	0.19 (0.04–0.87)	
	Dominant	T/T	186 (70.2%)	148 (74.4%)	1.00	0.320
		G/T‐G/G	79 (29.8%)	51 (25.6%)	0.81 (0.54–1.23)	
	Recessive	T/T‐G/T	252 (95.1%)	197 (99%)	1.00	0.012
		G/G	13 (4.9%)	2 (1%)	0.20 (0.04–0.88)	
	Overdominant	T/T‐G/G	199 (75.1%)	150 (75.4%)	1.00	0.940
		G/T	66 (24.9%)	49 (24.6%)	0.98 (0.64–1.51)	
	Log‐additive	—	—	—	0.74 (0.52–1.06)	0.100

Note

SNP: single‐nucleotide polymorphism; HWE: Hardy–Weinberg equilibrium; ORs: odds ratios; 95% CI: 95% confidence interval.

The GenBank reference of *IL‐4*: NC_000005.10.

*p*‐values were calculated by logistic regression analysis without adjustment.

### 3.3 Association between haplotypes in *IL‐4* and lung cancer risk

**Figure 1:**
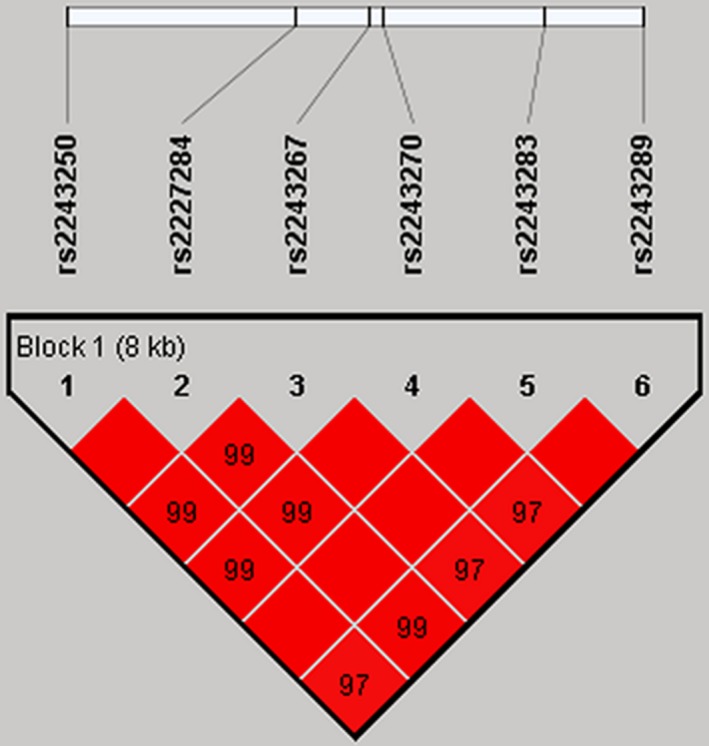
Linkage disequilibrium analysis for six SNPs in the logistic regression model. Standard color schemes indicate different levels of LD. Bright red: LOD > 2, D’ = 1

We further performed linkage disequilibrium analysis for six SNPs in the logistic regression model. The haplotype structure of the *IL‐4* gene was analyzed, and single LD block consisting of all six SNPs were detected (Figure 1). Four common haplotypes were listed in Table 5. Interestingly, we found that the C_rs2243250_G_rs2227284_G_rs2243267_A_rs2243270_C_rs2243283_A_rs2243289_ haplotype was associated with a significantly decreased lung cancer risk (OR = 0.63, 95% CI = 0.42–0.96, *p* = 0.034). After adjustment of age, the association was still significant (OR = 0.67, 95% CI = 0.46–0.97, *p* = 0.035).

**Table 5:** *IL‐4* haplotype frequencies and the association with the risk of lung cancer

	rs2243250	rs2227284	rs2243267	rs2243270	rs2243283	rs2243289	Freq	Crude analysis		adjusted by age	
								OR (95% CI)	*p* ^a^‐value	OR (95% CI)	*p* ^b^‐value
1	T	T	C	G	C	G	0.598	1.00	—	1.00	—
2	T	T	C	G	G	G	0.185	0.76 (0.54–1.08)	0.120	0.78 (0.53–1.14)	0.200
3	C	G	G	A	C	A	0.150	0.67 (0.46–0.97)	0.035	0.63 (0.42–0.96)	0.034
4	C	T	G	A	C	A	0.050	0.73 (0.40–1.33)	0.300	0.87 (0.44–1.73)	0.690
Rare	*	*	*	*	*	*	0.013	0.38 (0.10–1.45)	0.160	0.38 (0.08–1.68)	0.200

Note

OR: odd ratio; 95% CI: 95% confidence interval. The GenBank reference of *IL‐4*: NC_000005.10.

*p* ^a^‐values were calculated by Wald test without adjustment.

*p* ^b^‐values were calculated by Wald test adjusted for age.

* indicates the rare haplotypes with lower frequencies.

### 3.4 SNP–SNP interaction and lung cancer risk

MDR software package was used to detect the potential interactions between the analyzed SNPs in relation to lung cancer risk. Table 6 summarized the cross‐validation consistency and the prediction error of best models. Obviously, the three‐locus model (rs2243250, rs2243284, rs2243283) had a maximum testing accuracy of 56.4% and a maximum cross‐validation consistency (10/10) that was significant at *p < *0.05 level, after determined empirically by permutation testing.

**Table 6:** Predication of lung cancer risk factors in MDR analysis

Best model	Training accuracy (%)	Testing accuracy (%)	Cross‐validation consistency	χ^2^	*p‐*value
rs2243250, rs2243283	55.5	50.5	5/10	5.223	0.022
rs2243250, rs2227284, rs2243283	57.6	56.4	10/10	14.607	*p < *0.000
rs2243250, rs2227284, rs2243283, rs2243289	58.0	56.7	9/10	16.652	*p* < 0.000

Note

MDR: Multifactor dimensionality reduction. The GenBank reference of *IL‐4*: NC_000005.10.

*p*‐values were calculated by Pearson χ^2^ test.

## 4 DISCUSSION

In the present hospital‐based case‐control study, we investigated the association between six single nucleotide polymorphisms in the *IL‐4* gene and lung cancer risk in the Chinese male populations. Among these SNPs, rs2243250 and rs2243267 were associated with a decreased risk of lung cancer. In the genetic model analysis, rs2243250 decreased lung cancer risk under the log‐additive model, and the G/G genotype of rs2227284 conferred a protective effect on lung cancer susceptibility under the codominant and recessive models. Additionally, LD block was constructed with six SNPs in *IL‐4*, and we found that the CGGACA haplotype was related to a significantly decreased lung cancer susceptibility. In the result of MDR, the potential interactions among the three SNPs (rs2243250, rs2243284, rs2243283).

IL‐4 is a pleiotropic type Ⅱ cytokine which is encoded by the gene *IL‐4* located within the cytokine gene cluster on chromosome 5q31.1 (Rosenwasser et al., 1995). Multiple biological processes are regulated by *IL‐4*, such as proliferation, differentiation, and apoptosis in various cell types (Nelms, Keegan, Zamorano, Ryan, & Paul, 1999). However, the role of *IL‐4* in cancer is paradoxical. Okada et al. found that direct delivery of *IL‐4* gene to a central nervous system tumor site prolongs the survival of animals by inhibiting tumor growth to some extent (Villa, 2001). Lee et al. found that *IL‐4* exhibited a strong inhibitory function in the two critical steps of angiogenesis—migration of endothelial cells to the inflamed site and differentiation of migrated cells to the organized vessel structure (Lee et al., 2002). Based on the above evidences, it is suggested that IL‑4 has strong potential as a tumor therapy agent. But, there is still some evidence that *IL‐4* is a tumor‑promoting molecule. For instance, Stremmel et al. found that IL‑4 knockout mice are more resistant to tumor challenge than IL‑4 competent mice, indicating that *IL‐4* may play a critical role in abrogation of the antitumor immune response (Stremmel, Greenfield, Howard, Freeman, & Kuchroo, 1999). In addition, the expression of *IL‐4* was up‐regulated in many cancers, such as colon cancer, NSCLC, and cervical cancer (Al‐Saleh et al., 1998; Asselin‐Paturel et al., 1998; Berghella et al., 1998). Therefore, further studies are required to clarify the real function of *IL‐4* in cancers.

We found that rs2243250 was related to a lower risk of lung cancer. This was consistent with the previous studies. In 2012, Li et al. conducted a case‐control study in Chinese population and reported that frequencies of *IL‐4* –590 genotype (TC and CC), and −590 C allele were significantly lower in patients with NSCLC than in healthy controls (Li et al., 2012). The same year, Gomes et al. reported that increased expression of *IL‐4* associated with the TT genotype may contribute to immune surveillance during NSCLC development in Caucasian (Gomes et al., 2012). Besides, many studies showed that rs2243250 were associated with genetic susceptibility to other diseases, such as asthma, rheumatoid arthritis, and multiple sclerosis (Liu, Li, & Liu, 2012; Qiu et al., 2015). In our results, we revealed that rs2243267 and rs2227284, two less studied SNPs, were also found to be associated with lung cancer in this study. As well as, the relationship between rs2227284 and lung cancer risk has not been previously reported and we firstly reported it for the first time.

However, there were still some deficiencies in this study. Firstly, some bias in population selection should not be ignored. We observed the HWE in the controls, ORs, and 95% CIs on the basis of logistic regression analysis adjusted by age and gender to eliminate the bias to some extent. Secondly, the sample size was not larger and the reliability of the results needs to be further verified. Third, other risk factors (occupational exposures, physical activity etc.) were not included and should be considered to be assessed in the future and more comprehensive analyses about *IL‐4* polymorphisms in lung cancer are needed to confirm the results.

In conclusion, we demonstrated that rs2243250, rs2243267, and rs2227284 in *IL‐4* gene are associated with a decreased risk of lung cancer in northwestern Chinese males. These results may help improve the understanding of relationship between lung cancer and inflammation related gene *IL‐4*. On the other hand, the lung cancer‐associated molecular markers identified here might be useful as diagnostic and prognostic markers for lung cancer in future clinical studies.

## CONFLICT OF INTERESTS

The authors declare no potential conflicts of interest.
